# Differences in epidemic spread patterns of norovirus and influenza seasons of Germany: an application of optical flow analysis in epidemiology

**DOI:** 10.1038/s41598-020-70973-4

**Published:** 2020-08-24

**Authors:** Tabea Stegmaier, Eva Oellingrath, Mirko Himmel, Simon Fraas

**Affiliations:** 1 BMBF Junior Research Group BIGAUGE, Carl Friedrich von Weizsäcker-Centre for Science and Peace Research (ZNF), University of Hamburg, Hamburg, Germany; 2 Department for Microbiology and Biotechnology, Institute for Plant Sciences and Microbiology, University of Hamburg, Hamburg, Germany

**Keywords:** Ecological epidemiology, Image processing, Influenza virus, Viral infection, Policy and public health in microbiology

## Abstract

This analysis presents data from a new perspective offering key insights into the spread patterns of norovirus and influenza epidemic events. We utilize optic flow analysis to gain an informed overview of a wealth of statistical epidemiological data and identify trends in movement of influenza waves throughout Germany on the NUTS 3 level (413 locations) which maps municipalities on European level. We show that Influenza and norovirus seasonal outbreak events have a highly distinct pattern. We investigate the quantitative statistical properties of the epidemic patterns and find a shifted distribution in the time between influenza and norovirus seasonal peaks of reported infections over one decade. These findings align with key biological features of both pathogens as shown in the course of this analysis.

## Introduction

This work presents a novel perspective on the analysis of fine scale and high resolution epidemic datasets. We utilize optic flow, a method to observe and describe movement of patterns in videos, to analyze high resolution spatio-temporal epidemic patterns of noro and influenza seasons. (See Methods: Optic flow for details.) Infectious diseases are widespread in our country and history. Depending on the capability of the agent as a pathogen, its environmental stability and infection rate, a disease’s spread could result in an epidemic, limited to a community at a particular time or, in worst case, to a worldwide pandemic. Past epidemics and pandemics, like the Influenza 1918, lead to a population apprehensive of possible dissemination of infectious diseases resulting in an increased effort in modelling and surveillance of known existing communicable diseases. In the present time, infectious diseases as noro or influenza occur in periodic epidemics and pandemics with worldwide deaths of 290.000–650.000 per year for influenza^1^. In comparison 1.45 million deaths caused by acute gastroenteritis and 18 % of these infections were triggered by viral infections^2^. In Germany, influenza and noro infections are observed as the main communicable diseases^3^. However, there is a big number of non-registered cases in both illnesses due to people not seeking medical attention and treating the symptoms at home.

In the past there have been numerous views on influenza epidemic patterns^4,5^ and seasonality causes^6,7^. Other studies have targeted single^8^ or multiple regions^9^ in time-domain with pattern analysis of the epidemic data of influenza using lab diagnostics. Others have looked at regional influenza patterns utilizing the distribution of antiviral medication specific to influenza^10^. Seasonality of influenza can be regarded as a well-established concept. But is the spatial wave-like pattern of influenza outbreaks also well-established from historical data? And does the description of a wave hold true for another viral but profoundly different disease like norovirus infections? In literature as well as in our calculations we observed a certain seasonality of norovirus and influenza cases in Germany. In comparison, norovirus infections are examined in the winter month (Dec./Jan.) whereas influenza is monitored at the beginning of the new year (Jan./Feb.)^11,12^. We found a pattern which could be observed as an alternating transition from norovirus infections to influenza. Influenza occurs explosively in the population, whereas norovirus appears in very small numbers from time to time over the year, ending in a seasonality of accumulated cases in winter from December to January. In a yearly context, seasonality of norovirus can, as well, be regarded as a well-established concept^13^. We study the patterns of two winter-seasonal diseases with a combination of an innovative and established method to investigate a possible dynamic of interconnectedness. The different dynamics of two diseases associated with winter and wave-like incidence are an interesting object of study from the perspective of civil preparedness and possible prediction patterns. With the ability to identify and predict natural epidemic patterns we can work towards detection of unnatural patterns. The analysis of these naturally occurring patterns highlights possible distribution paths for potential future epidemic events regardless of origin. One of these winter-seasonal diseases is caused by an enveloped single-stranded RNA virus, the influenza virus. The influenza virus belongs to the Orthomyxoviridae family and is divided into A, B, C and D influenza virus strains, as well as further subtypes. However, only influenza virus strains A, B and C are known to infect humans^14^. Infections with virus strain D have not yet been observed in humans, but this strain does have the potential to infect human cells^15^. The transmission occurs from person-to-person, but also from animal-to-person and person-to-animal. The contagion usually happens via droplet infection and contaminated surfaces, rarely by contaminated food or water. For the transmission a droplet of $>5 \mu$m diameter is sufficient for infection, and by sneezing and coughing more than half a million of virus particles are spread^19–21^. Influenza causes severe infections of the respiratory tract with fever and lasts about 3–7 days^22^. The risk of contagion remains up to one week onset of the first symptoms while the environmental stability of influenza virus is up to 4 days in the environment, less than norovirus. In literature the mutation rate of influenza varies from type to type. The mutation rate of influenza A estimated at $7.1\times 10^{-6}$ to $4.5\times 10^{-5}$ substitutions per nucleotide per cell infection cycle and $2.7\times 10^{-6}$ to $3.0\times 10^{-5}$ substitutions per nucleotide per ssRNA strand copied per year^23^ influenza has a very high level of morbidity and mortality and therefore leads to challenges in effective public health surveillance—especially due to the good adaptability of the virus to its environment which complicates the development of long lasting vaccinations^24^.

**Figure 1:**
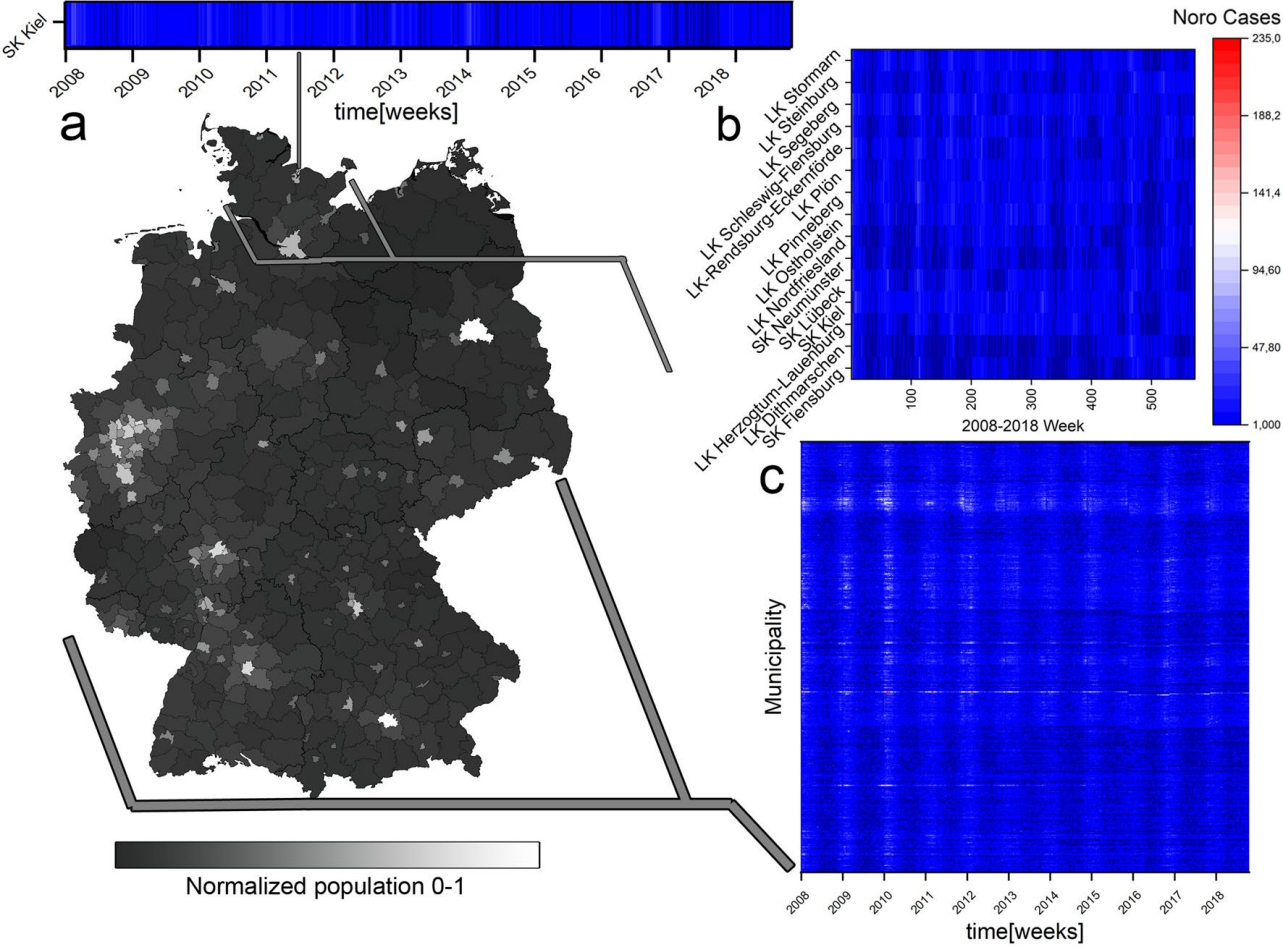
Overview of the spatial and temporal structure of the heatmaps and data base of this analysis. The map of Germany in NUTS-2 region resolution is tinted in greyscale according to normalized total population of municipality.(**a**) A single municipality cutout from the bigger heatmap showing the years 2008–2018 norovirus case count. (**b**) Heatmap of a single state (Schleswig-Holstein, northmost state of Germany) showing decade norovirus case counts. (**c**) The whole country heatmap for Germany over the years 2008–2018. Every pixel of these heatmaps represents a municipality in the vertical axis (in no particular order), a week in the horizontal axis (from 2008–2018) and the norovirus count level according to the colormap. Heatmaps in high resolution can be found in the supplemental data. The Germany map was created in SAGA Gis 7.6.1^16^ with publicly available data from the BKG Germany^17^. The heatmaps were created in Origin(Pro) 2019b^18^.

The second winter-seasonal disease we studied is caused by an non-enveloped single-stranded RNA virus, the norovirus, which belongs to the Caliciviridae family and is one of the most common causes of gastroenteritis. The transmission occurs from person-to-person by smear infections and contaminated food and water or surfaces^25^. Globally, there are 685 million norovirus infections per year with 70,000 to more than 200,000 deaths per year worldwide, mainly children in developing countries are affected^26–28^. Currently, six genogroups of noroviruses are known, which are subdivided into more than 30 genotypes. The genogroups I, II and IV are the relevant groups causing infections in humans. Thereby, genotype GII.4 caused most of the human clinical cases over the past decade and lead to six pandemics since 1995. In the meantime, the newly emerged genotype GII.P17-GII.17 became the predominant virus strain in some parts of Asia^29^. Interestingly, genotype GII.4 is more often associated with transmission from person-to-person, whereas strains of the genogroups I and other II are more often associated with food-borne transmission^30^. Human norovirus infection lasts about 12 to 48 h and the risk of contagion remains up to 14 days after infection. Already 10 to 100 virus particles are sufficient for infection and the virus is able to survive up to 140 days in the environment without a host^31–33^. Additionally, common RNA viruses reveal a high mutation rate ($$10^{-3}$$ to $$10^{-5}$$), human noroviruses exhibit an estimated mutation rate from $$1.04\times 10^{-3}$$ to $$6.10\times 10^{-4}$$ nucleotide substitutions per site per year, a very high rate in comparison to other viruses^34,35^. Especially GII.4 strains show a high rate of mutation and evolution, which could contribute to the frequent emergence of antigenically divergent strains^36^. Genotyping analysis showed that some GII.4 strains were able to cause widespread regional epidemics, but with a limited geographic distribution^37^. Clinical symptoms of the diseases include diarrhea, vomiting and fever, leading to severe dehydration and nutrient deficiency. However, there are also asymptomatic cases. These represent a particularly high risk because the infection goes unnoticed and the person is still contagious. Further, there is no vaccination available, only the symptoms can be treated^25^. Norovirus is very contagious and can pose a major threat to public health. The global economic burden of norovirus infections is thought to be in the range of $3.4 billion for health systems and $47.2 billion for societal costs^38^. Therefore, large-scale norovirus outbreaks could contribute to severe economic constraints. Furthermore, norovirus is considered as potential bioterrorism hazard due to its biological properties (e.g. tenacity, low minimal infection dose) and medical implications (e.g. acute and debilitating symptoms, no vaccination possible yet). Norovirus could therefore considered as Category B bioterrorism agent, according to the National Institute of Allergy and Infectious Diseases classification of pathogens and toxins^39^.

Enabling better understanding and facilitating ensures better preparedness for emerging threat situations. Understanding naturally emerging patterns to be able to identify potential abnormal patterns is vital. If there was a sudden increase of norovirus cases outside of the seasonal patterns we identify in this paper a qualified identification of unnatural incidents will be possible. For now this would of course be limited to the spatial scope of this study.

## Results

We analyzed influenza and norovirus infection reports from the decade of 2008 to 2018 gathered by the Robert Koch-Institute in the Survstat Database^40^ (see and Methods for details). We procured case numbers for all German municipalities (413 on NUTS (Nomenclature des unités territoriales statistiques—statistical regions) (3 level) present in the database as well as their respective metadata from the DE Statis and DE BKG databases^17,41^. The aim was to find evidence for wave-like distributed incidences and the dynamics of two common viral communicable diseases in central Europe. To prove that the observed patterns are not purely random we generated Monte-Carlo (MC) simulated artificial epidemic data with three different constraints. First, the possible case number was limited to the binned observed minimum (0) and maximum (235) per week. In the second constraint we limited the maximum time of the season to 10% of the weeks per municipality. And in the third constraint we limited the season to the weeks 0–14 (See Fig. 4 and “Methods” for details).

**Figure 2:**
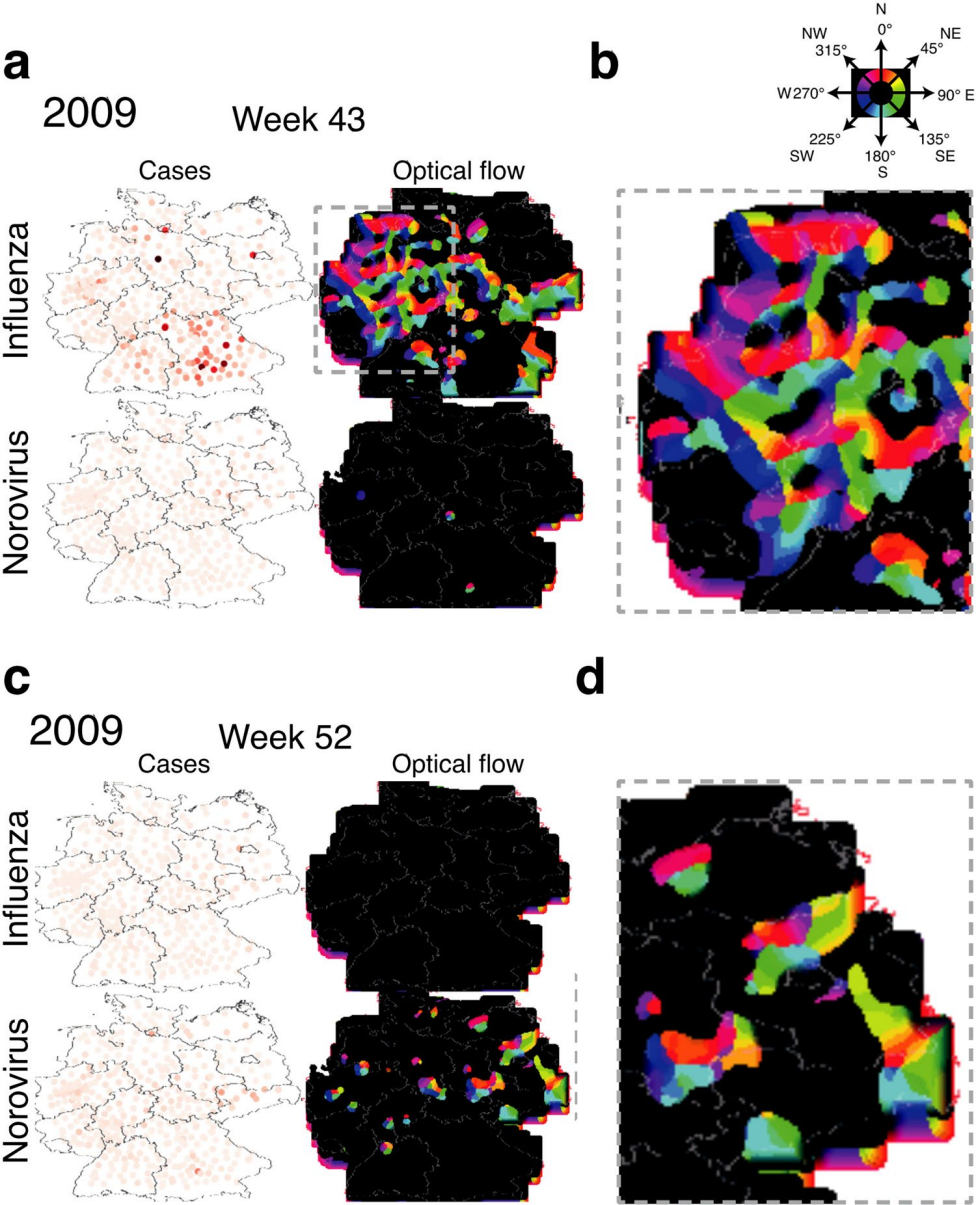
Examples of reported cases incidence and optical flow analysis for norovirus and influenza infections at selected time points. Dots on the incidence maps (white with orange-red intensity graded dots) correspond to the centroid coordinates of the reporting municipality. Light grey lines indicate state-borders. (**a**) Week 43 of the year 2009 shows a strong spread of influenza case reports to the north–west while there are high incidence numbers in the south. (**b**) A zoomed in view of the north-west area. (**c**) Week 52 of the 2009 season shows the peak optical flow activity for norovirus infection case reports with strong movement towards the east (**d**) and around individual areas in the west while incidence remains in the southern area. The federal state border overlay was created in SAGA Gis 7.6.1^16^ with publicly available data from the BKG Germany^17^.

### Overview of epidemic pattern of influenza and norovirus infections

In order to visualize the data and get an overview of the epidemic situation we created heatmaps (Fig. 1). These show the decade in horizontal axis and all German municipalities on the vertical axis (in no particular order) showing higher case counts of norovirus and influenza with brighter pixels (from blue over white to red) according to the legend. Thus the (vertical) patterns in these particular heat maps are of no significance as there is no spatial correlation in the vertical placement. Each pixel represents a week in a municipality for the decade 2008–2018. While norovirus shows no week that exceeds 235 reported cases in this data source influenza exceeds 235 reported cases 3876 times out of 235,914 datapoints. This mainly occurs in the peak season of 2018. To preserve the integrity of the histogram this was also put into the 235+ bin. For norovirus and influenza infections we see a wave pattern focusing on the winter months (Fig.7). For influenza this pattern is stronger pronounced than norovirus (see Fig. 2 for still picture and supplemental videos S1 and S2 for animation).

### Tempo-spatial distribution and quantification of influenza and norovirus waves

In order to identify a wave-like incidence pattern that also applies to the spatial distribution, we needed to grasp the movement of case-report patterns. For this we utilized the method of optical flow, an established concept from the field of computer vision (for details see Materials and methods—Optical flow) to identify moving objects or regions of similarity within an area. This was done for the incidence maps of norovirus, influenza and MC controls constraint 1–3 for the whole decade 2008-2018. Videos of norovirus and influenza incidence and optical flow can be found in Supplementary Video S1 for the whole decade, for the season of 2009-2010 in detail in Supplementary Video S2.

**Figure 3:**
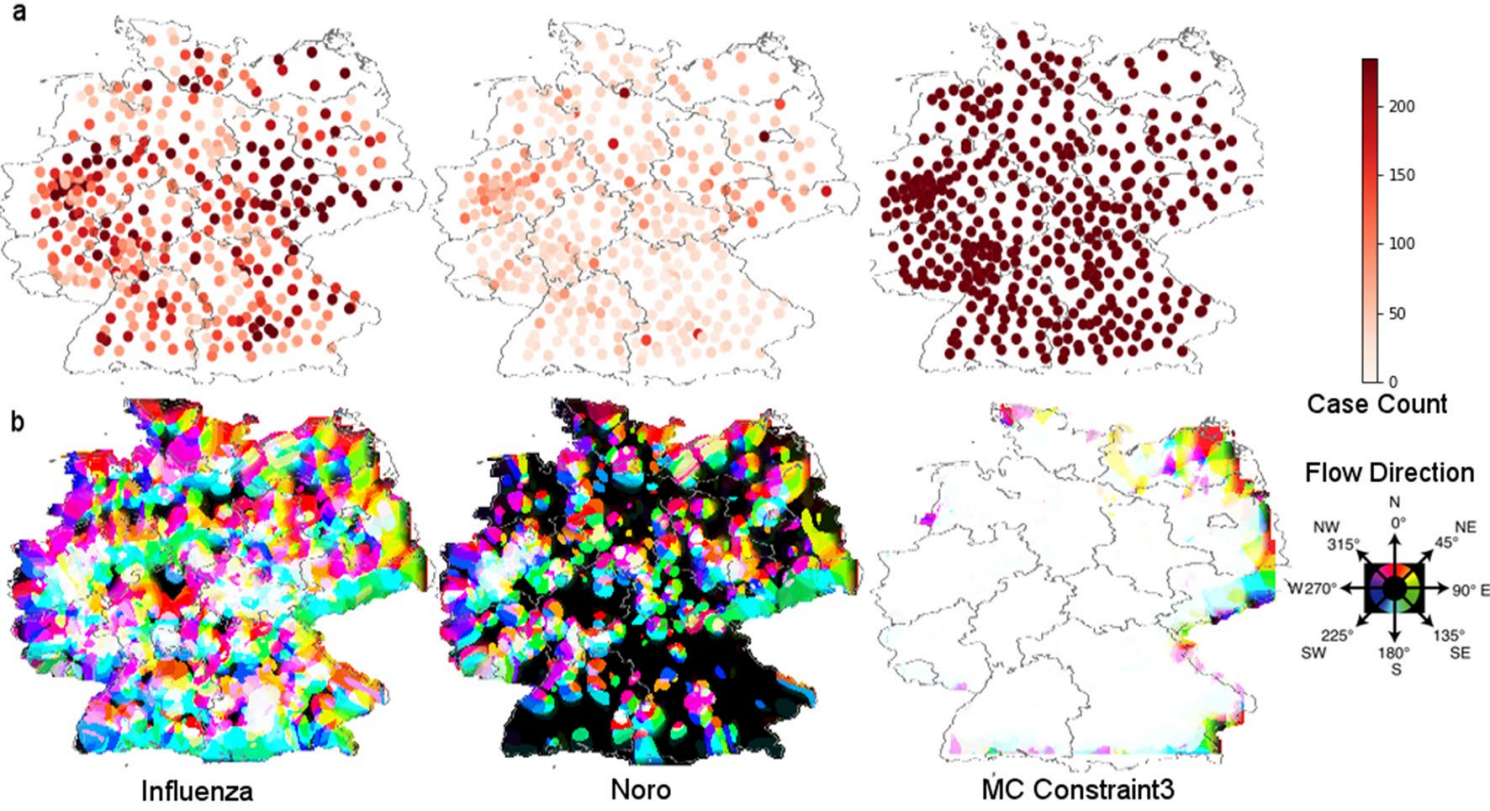
Summed up signals (maximum projections) of case counts and optical flow data over the decade of influenza, noro and MC Constraint 3. (**a**) The incidence maximum projections of influenza, norovirus and MC Constraint 3 show the summarized incidence rates for the decade with MC Constraint 3 showing maximum saturation. (**b**) The optical flow maximum projections for influenza, norovirus and MC Constraint 3 show the summarized optical flow activity for each with norovirus, influenza showing black areas of no activity in optical flow and MC Constraint 3 which shows maximum undirected flow. The federal state border overlay was created in SAGA Gis 7.6.1^16^ with publicly available data from the BKG Germany^17^.

The season of 2009–2010 (Fig. 2) showed a very high activity in incidence and optical flow probably due to the H1N1 epidemic occurring and serves as a prime example for the utility of the optical flow method as this particular season highlights the possible spatial paths of the epidemic wave very well. We keep seeing paths again and again during the decade. Looking at individual still frames the superior feature of the optical flow analysis versus pure incidence plot becomes evident (Fig. 3). For influenza we see a strong incidence in the south-east region while the optical flow clearly shows a strong transition from the city centers in the west to the north and south-east during this week indicated by the red and green markings stretching over the east. Whereas the incidence in the western area is not as pronounced as in the south during the same time period. Optical flow captures the movement of incidence report, its direction and intensity in one image or video, without the need to look up all the corresponding values. For norovirus infections the still image shows a lower and more blanketed incident report situation. The optical flow activity is limited to individual metropolitan centers and slightly more spread in the east section. A different week was chosen to show norovirus peak activity as the two epidemic events’ peaks do not occur at the same time. In order to evaluate the optical flow activity and incidence over the decade a maximum intensity projection (Fig. 3) was done. The upper row (a) shows the summarized incidence over the decade revealing a difference in case numbers between influenza and norovirus infections with norovirus still showing strong incidence in certain high population density areas. The MC simulation shows a maximum incidence for all locations since the upper limit was the highest observed incidence count. The optical flow maximum projections (b) show that influenza waves cover the whole area over the decade, while norovirus optical flow activity is reserved to localized clusters with north–south movement as the dominant direction throughout and a west-bound tendency in the western part of Germany. Influenza is showing 339 no activity pixels and norovirus 10683. The MC constraint 3 control displays maximum activity throughout the decade as there are no rules/effects in the simulation data governing the flow. The simulation shows that by Monte-Carlo case-number distribution alone no pattern of movement can be extracted by optical flow (even more so in the videos).

**Figure 4:**
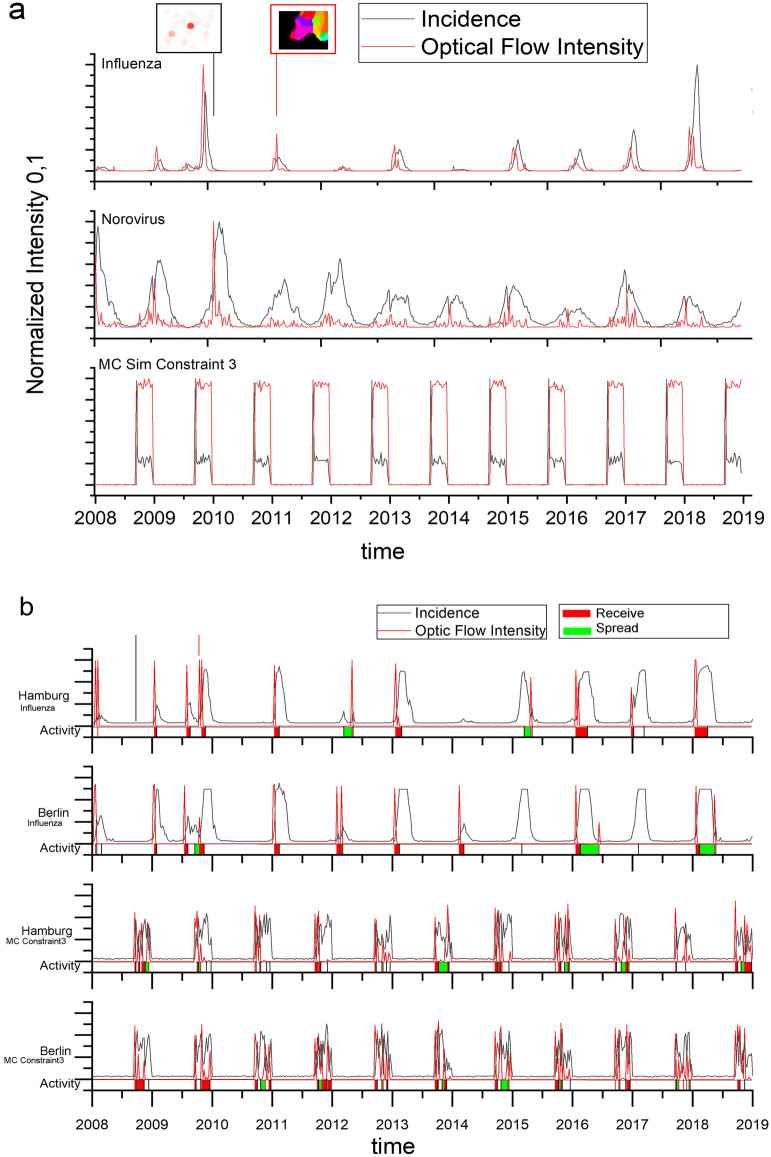
(**a**) Plot of the normalized optical flow and incidence intensity for the decade in influenza, norovirus and MC Constraint 3 control epidemic activity. (**b**) Plot of optical flow activity and Incidence in a normalized (0 to 1) range with Receive (red) and Spread (green) markers. For the city of Hamburg we see a spread pattern only in 2012 and 2015. For Berlin with more neighbors the dynamic is more complex. For both municipalities the analysis was repeated with MC simulation data resulting in an inconclusive pattern without clear activity markers for each year. Time scale: plotted are reported cases per week in the time frame shown.

The incidence sums and optical flow intensity was normalized and plotted (Fig. 4a). We see a pronounced wave pattern of incidence for norovirus and influenza which translates into optical flow signals very well for influenza and less so for norovirus infections. Influenza spreads in a globally pronounced pattern throughout the country whereas norovirus spreads in more geographically localized clusters showing no clear spread-receive patterns in the context of direct neighborhoods in the observed data. While the intensity plot for norovirus shows a noisy peak formation in comparison to rather defined peaks of influenza cases the different spread patterns can be clearly discriminated in the maximum projection (Fig. 3b) with a spotty coverage amounting to more zero pixels for norovirus than for influenza. Observing the dynamics of optical flow and incidence patterns allows to gain an informed perspective whether a point of interest (in this case two cities) receives or spreads in the epidemic context (Fig. 4b). For example, the city of Hamburg has six neighbors and shows a well identifiable pattern of disease spread and receive activity. For the city of Berlin having nine neighbors the spread pattern shows more fluctuations within one season. In the control data-set both show very convoluted patterns as all municipalities get random MC data.

**Figure 5:**
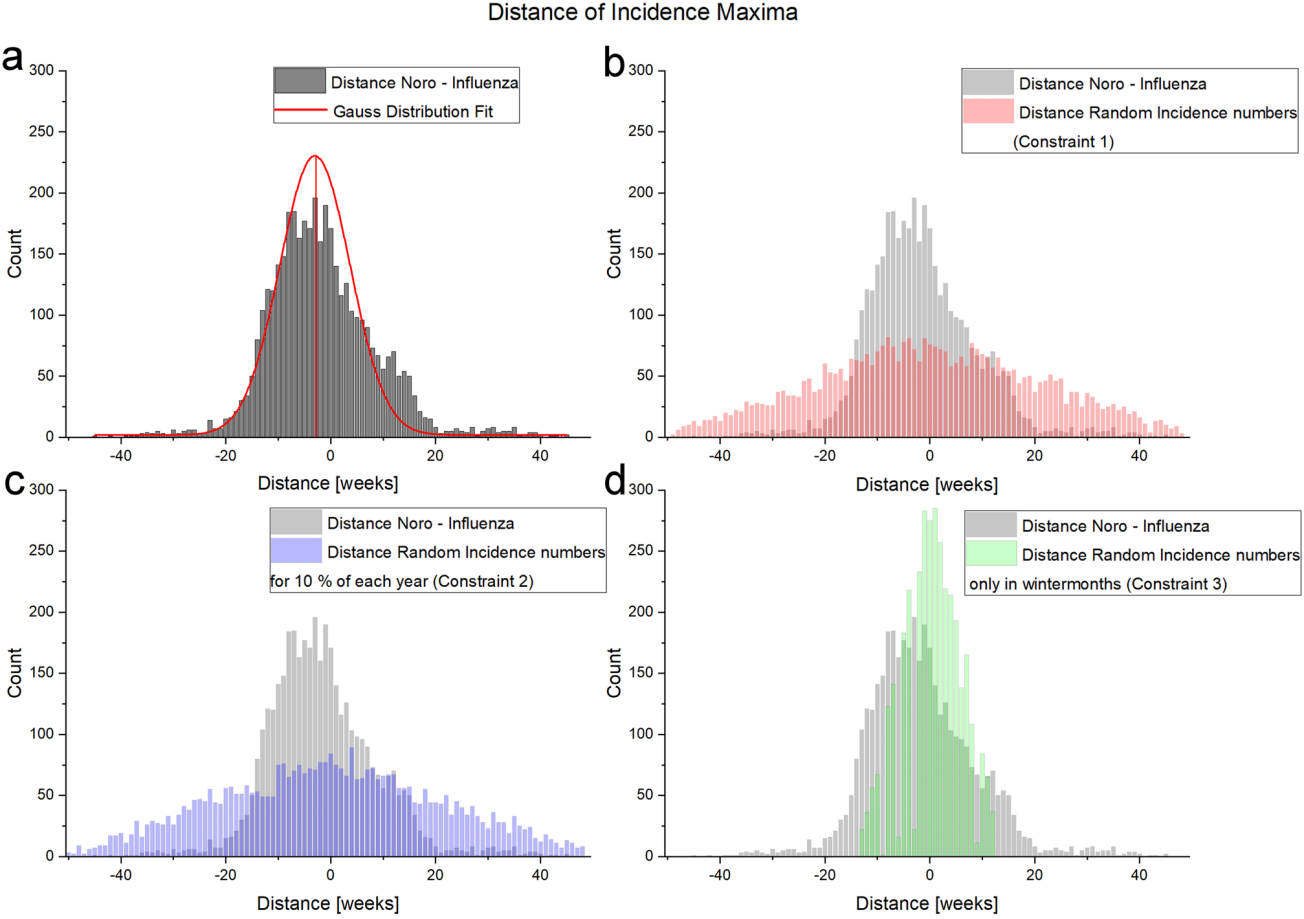
Analysis of the distances of incidence maxima of norovirus, influenza and the respective MC controls. (**a**) Incidence maxima distance count of real-world norovirus/influenza epidemic peaks. A normal Gaussian distribution with a shift of mu $=-3 (+- 0,24958)$ an a sigma $=6,724$ was fitted (red curve). The real-world maxima were plotted with the incidence maxima of the different constraints of MC simulation distances. (**b**) With MC constraint 1 (random incidence numbers) vs real data, (**c**) MC constraint 2 (random incidence numbers 10 % o the years) vs real data and (**d**) MC constraint 3 (random incidence numbers only in winter months) vs real data.

### Dynamic relation of norovirus and influenza waves

Regarding the distance of the seasonal peak between both infectious diseases for all municipalities together, we found, for the data available to us, a normal Gaussian distribution with an expectation of mu $=-3 (+- 0,24958)$ and a sigma $=6,724$ (Fig. 5). This indicates that, based on our data set, there is a 3-week gap between the incidence maxima of reported norovirus and influenza infections. Of note, this was not the case in any of the MC constraint data-sets. Especially the constraint 3 data-set shows a strong difference between random peak data and real data.

## Discussion

The application of optical flow analysis on complex epidemiological data-sets reveals infectious disease spreading patterns of two illnesses that are highly relevant for the public health sector in Germany. By using this method we could clearly show the wave-like spread pattern of influenza. Furthermore, we could discover a statistical temporal connection to norovirus infection cases despite the different norovirus spread pattern. For doing so we analysed publicly available source data of very high resolution in the spatial and time domain (Fig. 1). In the spatial domain the resolution scales down to municipalities and city states representing all 412 administrative regions of this level in Germany. In the time domain numbers for reported cases of confirmed norovirus and influenza infections are available for 573 weeks (with 2008, 2012 and 2016 being leap years). For the analysis we used reported norovirus infections and influenza cases. We excluded influenza-like illnesses (ILI), because we wanted to rely on infection disease cases confirmed by laboratory diagnostics. The distribution of time between norovirus and influenza infection peaks (Fig. 5) may seem counter-intuitive at first. Especially the optical flow data show a wave-like spread pattern for influenza seasons (Fig. 4) and no wave pattern for norovirus outbreak seasons. This can be interpreted as evidence for the very different nature of spread-mechanics between these two illnesses. In the southern parts of Germany we see a lot of incidences but a rather low geographical movement of both infection diseases in the optic flow. This effect is more prominent for norovirus infections (see Fig.3b) than for influenza.

The Gauss-resembling distribution underlines that we look at a very top down kind of data with a vastly complex system underneath. Germany offers an interesting spatial dynamical insight in infectious disease spreading because the country has a high population density of 238.5 people per square-kilometer compared to the western European countries with 179.5 and the EU at large with 33.7 people per square-kilometer. In-country flights in Germany with 23.5 million flight passengers^41^ or in France with 13.6 million passengers is rather low compared to much larger countries such as the USA reporting about 780 million in-country flights in 2018. In Germany long distance travels rely more on fast trains, car and buses. Public transport is of high relevance for travelling to outskirts of cities and between smaller towns, whereas travelling by car is of importance for remote areas in Germany. Europe and especially Germany feature a high rate (65% between 5–50 km) of regular travel to work of employed population of 45.1 million (as of the 2016 microcensus— DE Statis 2019)^41^ by public transport (non-plane). Unlike other approaches with modeling spatial transmission^42^, our method does not require graph inputs or information about underlying networks offering a more hands-on approach for governmental agencies and other actors in the public health sector. Other research groups have looked at seasonal influenza patterns^43^ using graph based calculations. In comparison the German influenza wave data shows a slightly different structure eluding the definition of a hard onset. A norovirus infection lasts only 48 h and thus quickly subsides again. However, it has a very high stability with a survival rate of 140 days. The under-reporting of cases poses a challenge in monitoring the spread of this infectious disease. Norovirus has a very high environmental stability and can tolerate extreme temperature fluctuations which ensure their survival on contaminated surfaces^44^. The possibility of contamination can be reduced to a minimum by thorough cleaning and disinfection because these viruses are transmitted via smear infections^45^. Especially young children and elderly people are affected by the infections. Because the immune system is not yet fully developed, children quickly become ill^46^. In elder people, the infection is often caused by contaminated food^47^. Due to the weakened immune system, they are particularly at risk here. Norovirus infections occur rather sporadically over the year: during the winter season increased numbers of infections are frequently reported. People are getting closer together because of the weather and holiday season. Heated apartments may act as incubator for this virus and other infectious agents and the human immune system may get weakened due to vitamin deficiencies^6^. Altogether, this increases the risk of infections and disease spreading. There are several circulating genotypes of human-pathogenic noroviruses known. Currently, the genotype GII.4 is predominant, but recent studies show, that in children multiple genotypes co-circulate with low to high incidence. Especially, genotypes GII.3, GII.6 and GII.2 together with GII.4 are able to cause norovirus infections in children under 5 years of age^35^. This phenomenon might contribute to the very different outcomes of regional norovirus outbreaks. Depending on the capability of GII.4 sub-strains to get transmitted to adults which usually have a higher geospatial mobility than younger children, regional spreading of norovirus might increase. For influenza, the impact of different circulating viral strains on the geospatial outbreak pattern is discussed below.

Influenza virus particles, on the other hand, do not have such high environmental stability, but are readily transmitted by droplet infection. Therefore, it may be sufficient enough just to be in the same room with an infected person to contract the disease. Due to the highly efficient aerogene transmission of influenza and the rather long time period of one week within an infected person may transmit the pathogen influenza spreading in human populations can increase exponentially with a high growth rate of infections. This mainly affects age groups in the middle range (30–50 years). Infected people may become symptomatic or even asymptomatic carriers for the disease and contribute to a wide spread of the infections over several days^48^. The increasing humidity and low temperatures in during the winter season promotes the transmission of influenza.

Spread pattern of different influenza virus strains dominating during flue season in Germany can be clearly discriminated by optical flow analysis. In a recent report, the German national public health institute highlights four unusual influenza seasons in Germany: 2009/10, 2012/13, 2014/15 and 2017/18^49–52^. We analyzed the corresponding published data on reported cases by the optical flow method. In 2009, a newly emerging pandemic influenza A strain H1N1 hit an immunological naïve population in Germany (Fig. S4 (a–h)). In the optical flow plots we observed a very distinct influenza spread pattern of this pandemic virus compared to other influenza seasons in Germany: high incidence values for influenza infections are going along with a wide spread of optical flow signals across Germany (Fig. S4 (d) and (h)). This pattern could be detected for the two distinct influenza A(H1N1) spreading events (2009, calendar weeks 24–30 and 35–53) described in the report by the German public health institute^49^, but was more prominent in the second outbreak wave during the winter season (Fig. S4 (h)).

The optical flow signals for influenza seasons 2012/13 and 2014/15 are characterized by less geographical spreading across Germany. In 2012, an equal distribution of influenza A(H1N1)pdm09, influenza A(H3N2) and influenza B virus strains was observed (Fig. S3 (a–d)), whereas in season 2014/15 the influenza A(H3N2) strain dominated over influenza A(H1N1)pdm09 and influenza B (Fig. S2 (e–h))^50,51^. A genetic drift in influenza A(H3N2) at the early beginning of the epidemic resulted in more infections most probably due to ineffectiveness of the vaccine used. Interestingly, the influenza season 2017/18 again showed high case numbers, but also an unusual age distribution among infected people in Germany^52^. In relation to the previous influenza outbreaks shown here more infections of people in age groups with a presumably higher mobility were reported in 2017/18. In the optical flow analysis, this season is characterized by increased regional spread of reported influenza cases on the geospatial-temporal scale (Fig. S2 (a–d)). Furthermore, optical flow activities run in front of reported maximum incidence values. In maximum intensity projections of optical flow data of selected calendar weeks we could observe clearly distinct spread pattern for the different influenza outbreak seasons analyzed here: the pandemic influenza outbreak in 2009 is characterized by an optical flow pattern covering most parts of Germany (Fig. S4), whereas the other seasons show a more fragmented pattern (Fig. S2 and S3). Of note, the unusual influenza outbreak 2017/18 shows a broader coverage of some parts of Germany placing this optical flow pattern (Fig. S2 (c)) closer to the pandemic event in season 2009/10 (Fig. S4 (g)).

While analysing both infectious disease spread pattern a question arose: Why is there an apparent connection between the spread pattern of norovirus and influenza infections on the time scale? One explanation for this phenomenon might be a negative influence of subsequent infections by both pathogens on the human immune system. Norovirus infections could lead to nutrient deficiency by severe diarrhoea and dehydration. Low light conditions and a poorer supply of nutrients cause, for example, a decreasing vitamin D level which leads to a weakened immune system^53^. Furthermore, norovirus infections mainly affect children and elderly people, because their immune system is not yet as good or not as well developed as a middle-aged person. Due to the incubation period of influenza, the infection is not noticeable at first. So many already infected people may still go to work and spread the disease^54^. In addition, the risk of getting infected persists even after the symptoms have subsided within a period of 7–10 days. This leads to the assumption that norovirus infections may support the spread of influenza. First, the human body is weakened by a norovirus infection which result in fewer nutrients and dehydration. In addition, there are lower temperatures, poor light conditions and a possible resulting vitamin D deficiency. Over time an increased number of infected people appear in the population, who despite of influenza symptoms or as asymptomatic carrier go to work and visit friends and family which leads in the end to an explosive spread of influenza infections throughout the population. As a key case we pay special attention to the distribution of 2009 where we could observe a major rise of both infectious diseases. In 2009/10, an increased number of noroviridae of genotype GII.4 with 3 different drift variants occurred predominantly in the German population^55^. Additionally, since season 2009/10 the number of genogroup GI strains circulating in Germany increased. Genotype GII.4 strains were causing 69% of all norovirus infections in winter season 2009/10, whereas in 2014/15 only 38% could linked to this genotype^56^. For both genogroups it is important to note, that the number of detected recombinant viruses (e.g., GII.6/GII.P7 or GI.6/GI.b) also increased. In the other case, in spring of 2009, an influenza pandemic occurred due to the influenza A subtype H1N1, also known as swine flu, which appears first in Mexico^57^. The interesting fact here is that in 2010/2012 a new norovirus strain occurs after the pandemic of influenza A H1N1^58^ Due to the high number of cases, modelling patterns can already be developed and analysed here in particular. For the season of 2009-2010 the peak epidemic activity for both illnesses (Fig. 5) Optical flow analysis of epidemiological data can be conveniently used to simplify complex disease spreading patterns as shown here. This would also allow the detection of unusual infectious disease outbreaks, if signatures of such an event could be observed in the visualised data set^59^. Furthermore, the investigation of recent disease outbreaks by optical flow analysis could help to understand spatio-temporal disease spread patterns in relation to existing or vacant medical resource in a better way. The results obtained could be used to improve resource allocation on a sound scientific basis. Both elements would contribute to strengthening of the public health system and would also enhance resilience of a society against unusual biological threats. For the analysis presented here, we could have used a graph like other research groups did. But the very high number of municipalities in combination with the dense neighborhood clusters and the timescale analyzed requires a lot computation time. Therefore, we decided to simplify the data into videos and take advantage of already established highly efficient image analysis algorithms to conduct our analysis. This method works without deeper knowledge of the connections of the underlying places. Epidemiological movement patterns can be discerned directly from the data and coordinates of occurrence. By this approach, the comparison of results gained from methods that include these information to gain new insights in previously unknown infection spreading routes. Advantage of the presented method is that it could be used to analyze spread patterns of any illness as long as coordinates and regularly timed incidence data for the locales is available (for example Covid-19). It requires no preconception of how an illness works.

In the cases analyzed here we see that norovirus infections clearly clusters around densely populated areas (compare Fig. 1 and Fig. 3) while the lower-density population areas are generally being left out by immediate neighborhood disease spreading waves. Reported influenza cases however do roll over lower-population dense areas in most years (see Videos^60^). However, incidence numbers are accumulated for each municipality, so intra-municipality resolution patterns are not represented in the findings discussed. We need a more detailed picture, especially in the case of circulating norovirus genotypes to deal with potentially emerging epidemic situations. This includes an improved reporting of laboratory-confirmed cases in order to provide a better coverage of norovirus infections. In the future, it might become possible that innovative data interpretation methods can be combined with anonymized movement profiles (e.g. derived from cell phones) to further elude low-level distribution of infectious diseases^61^. This could then validate our findings. A disadvantage of the method is the strong dependence on incidence data. This data has to be very precise as errors or postponed updates will be represented as part of the pattern. This post-event analysis cannot replace or visualize the tracking of individuals regarding infection chains during an epidemic. Ultimately, we show here that a better case-report system also enables a solid epidemiological pattern analysis by itself without the need for integrating individual movement profiles on a large scale. This could prove beneficial in cases, where data security (e.g., protection of personal and private data) is critical. Optical flow activity proves to be a valuable tool for a condensed view of the spatial distribution (Fig. 3) of reported influenza and norovirus infections.

## Conclusions

Our study shows a clear temporal and spatial wave dynamic for influenza. And a hotspot-focused dynamic for noro. This is achieved by employing imaging-derived method of optical flow together with classic tools of quantitative epidemiology. Our findings allow for a better understanding of disease-spreading as optical flow manages to set a series of many events (reported cases) into a common context. We provide an intuitive insight into highly complex epidemiological dataset accessible to peers in the area of epidemic study and beyond.

## Methods

### Data sources

Incidence report data on norovirus and influenza infections was acquired from the public Survstat database of the Robert-Koch Institute Germany^40^. In the whole body of this study we use total number of reports instead of pre-calculated cases per 100.000. Data for the decade from 2008–2018 was obtained spanning over 573 weeks. For the heatmap visualization case report data was fit into bins of 7.8 width and a maximum of 235+ bin where all weeks with more than 235 cases (0 for norovirus and 3876 for influenza out of 235,914 data points) ended up in. This was done in Origin(Pro) 2019b^18^. Coordinates and metadata for the 413 report municipalities (on NUTS-3 level) was acquired from the German Federal Agency for Cartography and Geodesy^17^. From this database centroid coordinates of all German municipalities were extracted and used to create the incidence map image stacks. We also used SAGA Gis 7.6.1^16^ to generate bitmap overlays of the borders of the federal states for orientation purposes.

### Imaging and optical flow

Incidence plots were generated with a jupyter notebook based script (with Matplotlib 3.1.1 in Python 3.7^62^). From these images stacks were created with Multi Stack Montage Plugin (Burri and Guiet 2015)^63^for ImageJ FIJI. Image analysis, optic flow analysis and preparation of figures was done in ImageJ^64^ with the Gaussian MSE plugin in the distribution FIJI^65^. Optic flow (Parameters: 8 px Neighboorhood, Sigma 4) on histogram stretched XY incidence plots of norovirus and influenza infections were compiled into an AVI movie (see supplemental data) and singular frames. This limits the resolution to the level presented here, as each pixel represents data like a heatmap does. Optical flow is an analytical approach to detect moving objects in videos or image sequences (for example a time series of microscope images). Here, this method is applied for the visualization of infectious disease spreading projected on a geographical map (for further reference see Agarwal et al 2016^66^ and Fortun et al 2015^67^).We utilize optical flow analysis to detect the direction and intensity of possible shifts of case numbers of norovirus and influenza infections based on an intensity plot (done with Python MatplotLib). An intensity plot for every week results in 570 frames that are analyzed as a stack in ImageJ Fiji with the Gaussian Window MSE Optic Flow plugin. This plugin analyzes the stack of images by taking pairs of images and comparing each pixel within a Gaussian kernel. The resulting output are frames (see Fig. 2) that show a black silhouette (the search radii for each detected data point) of the German municipalities on a white background (no data). The vector of infectious disease spreading is indicated by the color of the trace according to the RGB color wheel (see Fig. 2 or Fig. 3). The intensity of the movement of reported infections is indicated by the brightness of the color traces. Since historical data (0–235 cases per week per municipality) falls within a 0–255 range no scaling was necessary to stay within 8-bit range of the imaging analysis used. Contrast was enhanced by stretching the histogram range up to 255 grey values in figures showing here in Photoshop CS5 to ensure that all pixel are visible when printing this article on paper. Red-colored no-data areas in the original output images were re-colored here in white for better visualization. Of note, no data was dropped in the process and no over-stretching of the histogram occurred. For better orientation country borders were added as overlay after image processing.

### Monte Carlo simulation and data analysis

The control data-set is a randomly generated array of time and location independent incidence values between 0 and 235 over the time-period analyzed. This control was performed in order to prove that optical flows of historically observed infectious disease spreads are clearly distinguishable from random spreading patterns. Three different sets of randomized data were generated with different constraints in each data-set.

- First constraint: 0–235 cases per week, per municipality. (Observed case counts between 2008-2018)
- Second constraint: 0–10 cases in 90% of the weeks and 11–235 cases in 10% of the weeks
- Third constraint: Set only the winter weeks 0–14 per year per municipality to high case count (11–235), leave the rest at 0–10 cases

**Figure 6:**
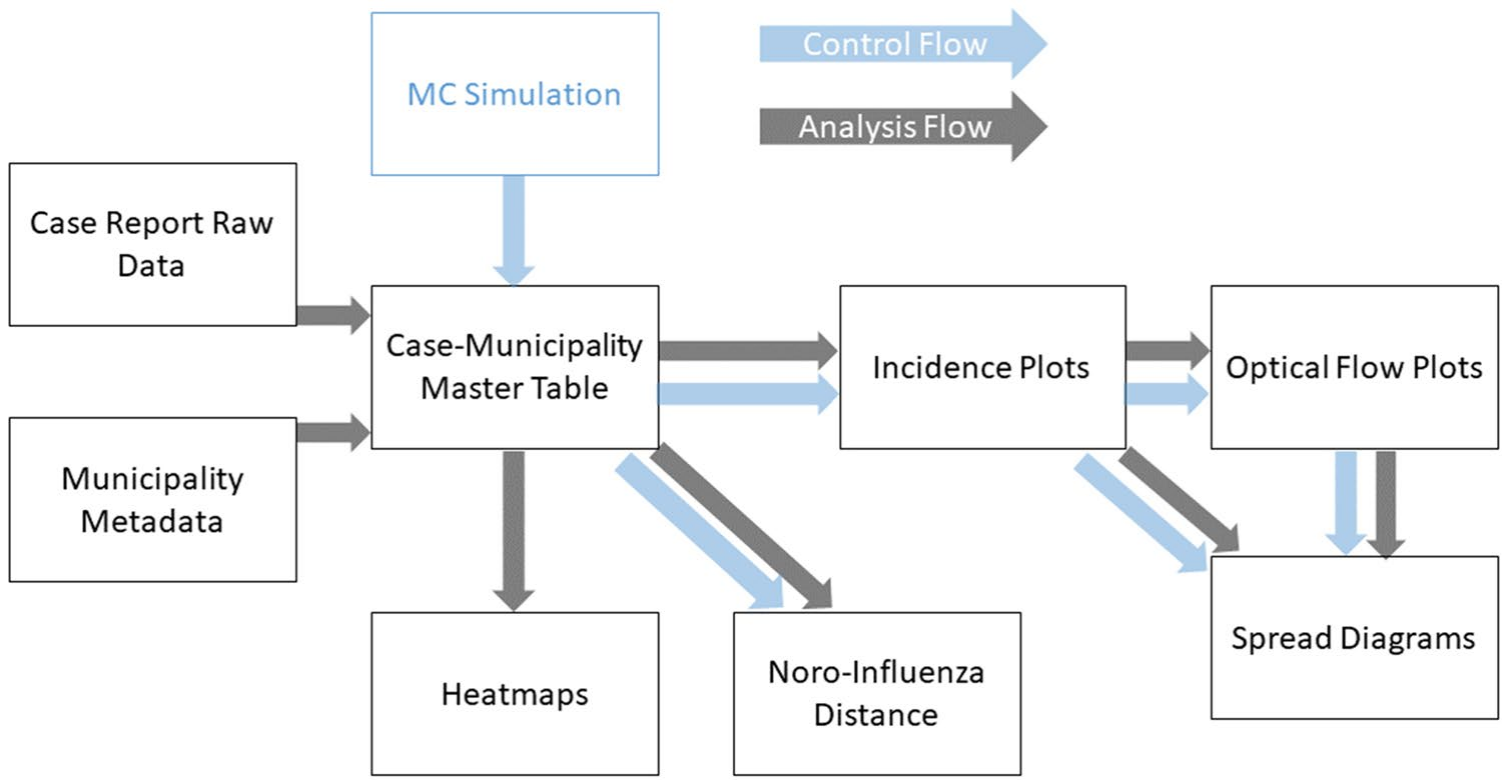
Workflow used by the authors to analyze the incidence datasets and generate optical flow data and derive information from it presented in this study.

Peak detection classified highest occurring peak of incidents as seasonal peak per year for the analysis of historical and control data. Data analysis was conducted by following a process (Fig. 6) that included MC control data from the earliest time point on. Case report data and municipality metadata was integrated into a master table from which heatmaps, distance data and incidence plots were generated. From the incidence plots optical flow plots were generated.

**Figure 7:**
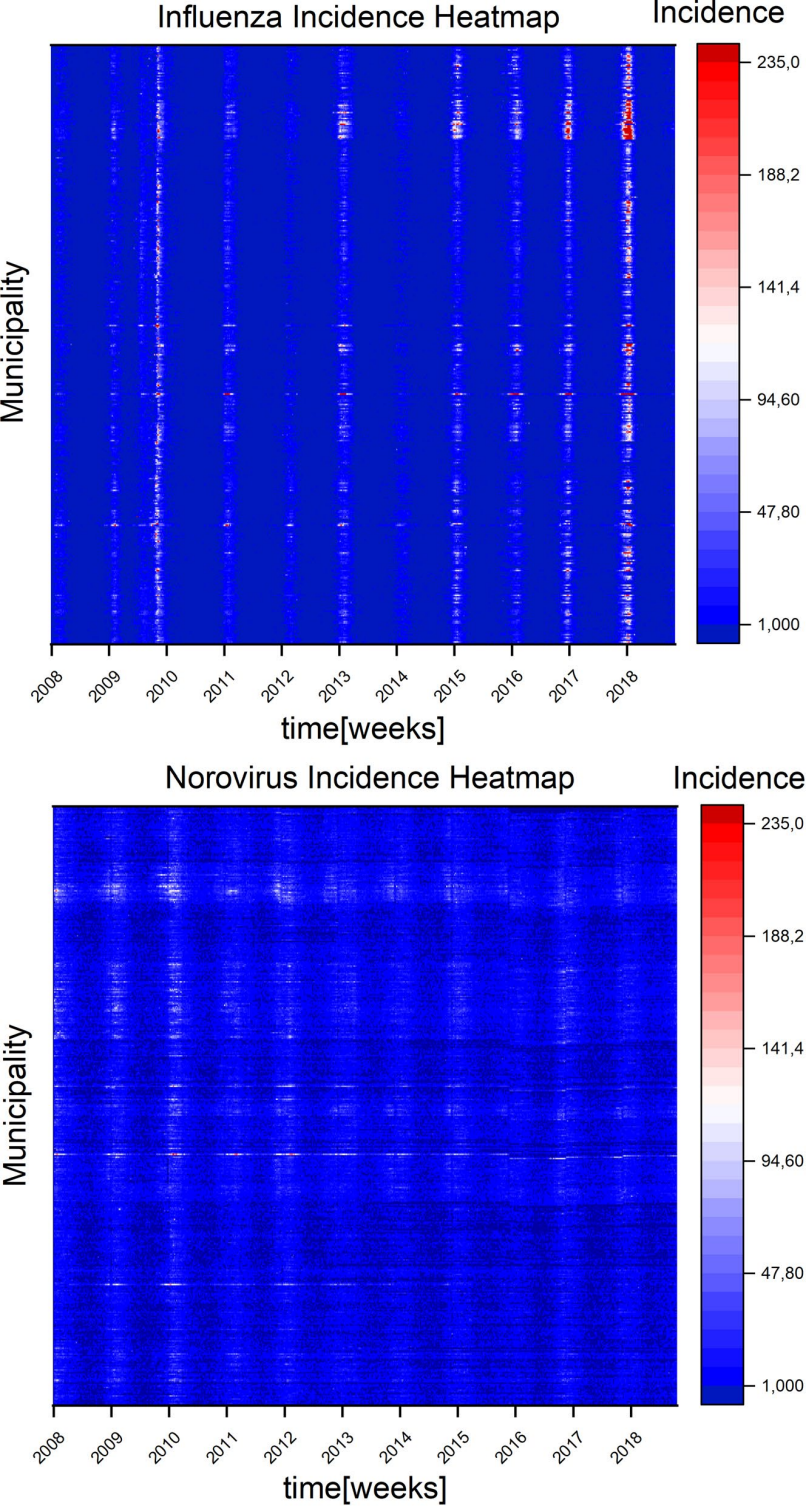
Heatmaps of influenza and norovirus case reports for all municipalities from 2008 to 2018. Every pixel represents a week’s report with the magnitude indicated by the color scale on the right. The heatmaps were created in Origin(Pro) 2019b^18^ with the contour-heatmap function.

## Supplementary information

Supplementary Video 1.

Supplementary Video 2.

Supplementary Information.

## Data Availability

All data cited in the manuscript and listed in the bibliography section is available by public access. The supplemental videos S1 and S2 have been uploaded to *Figshare* as alternative access method^60^.
