# Supplementary material for: Differences in epidemic spread patterns of norovirus and influenza seasons of Germany: an application of optical flow analysis in epidemiology
Source: Sci Rep. 2020 Aug 24;10:14125. doi: 10.1038/s41598-020-70973-4 (PMC7445178; doi:10.1038/s41598-020-70973-4)
Supplement: Supplementary file 3 — Supplementary Information. [file 41598_2020_70973_MOESM3_ESM.pdf]

Supplementary Information

Differences in epidemic spread patterns of norovirus and influenza seasons of Germany - an application of optical flow analysis in epidemiology

Tabea Stegmaier[1,*], Eva Oellingrath[1,2,*], Mirko Himmel[1,2,*], and Simon Fraas[1,+]

[1] University of Hamburg, Carl Friedrich von Weizsäcker-Centre for Science and Peace Research (ZNF), BMBF Junior Research Group BIGAUGE, Hamburg, Germany

[2] University of Hamburg, Institute for Plant Sciences and Microbiology, Department for Microbiology and Biotechnology, Hamburg, Germany

[*] These authors contributed equally to this work.

[+] To whom correspondence should be addressed: Simon Fraas simon.fraas@uni-hamburg.de

1 Supplementary Figures

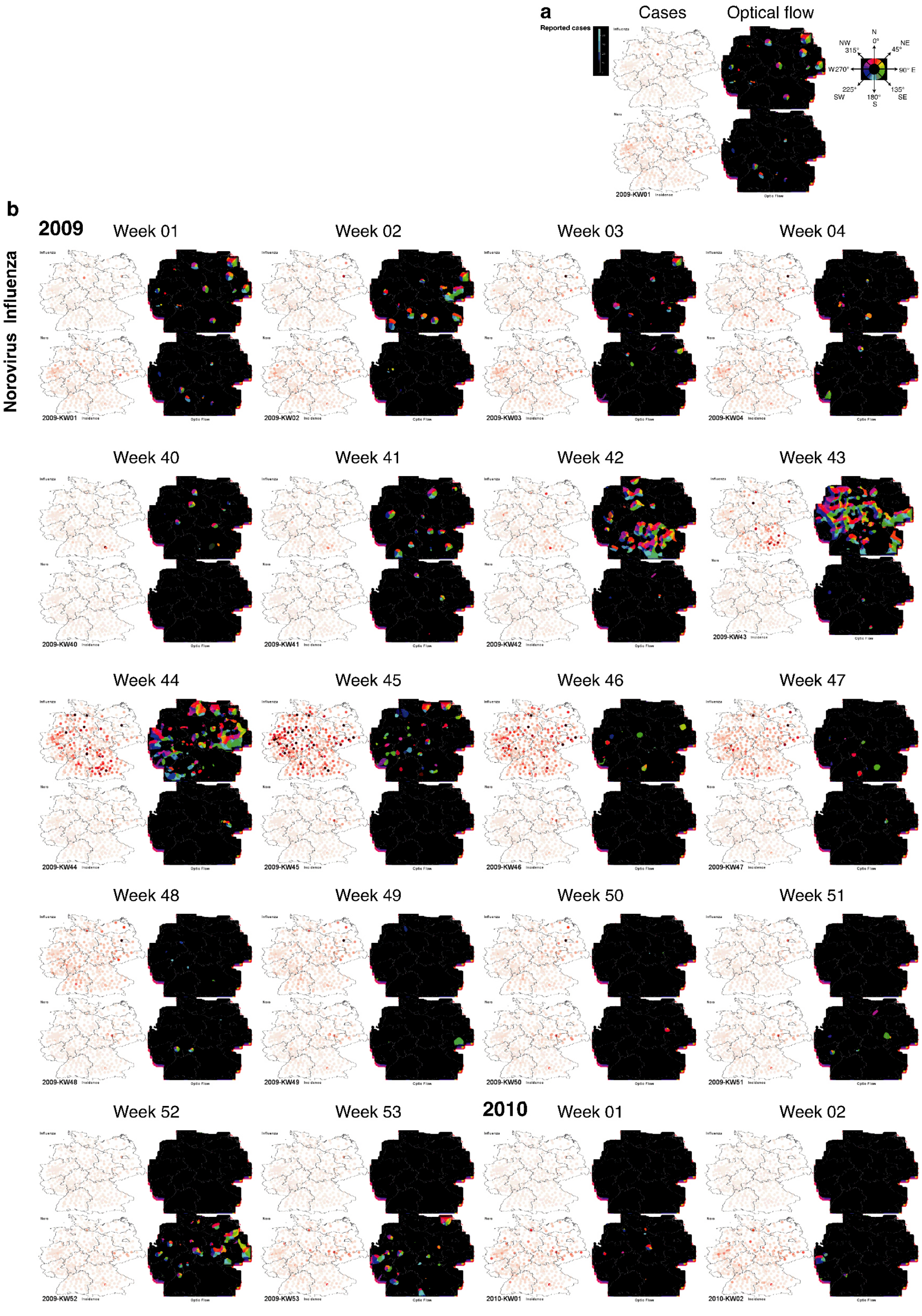

Figure S1: Selected still frames from videos S1 and S2. **(a)** Scaling of incidence reports and color code for geospatial movements. **(b)** Tempo-spatial distribution of influenza and norovirus incidence reports. The federal state border overlay was created in SAGA Gis 7.6.1 [6] with publicly available data from the BKG Germany [5].

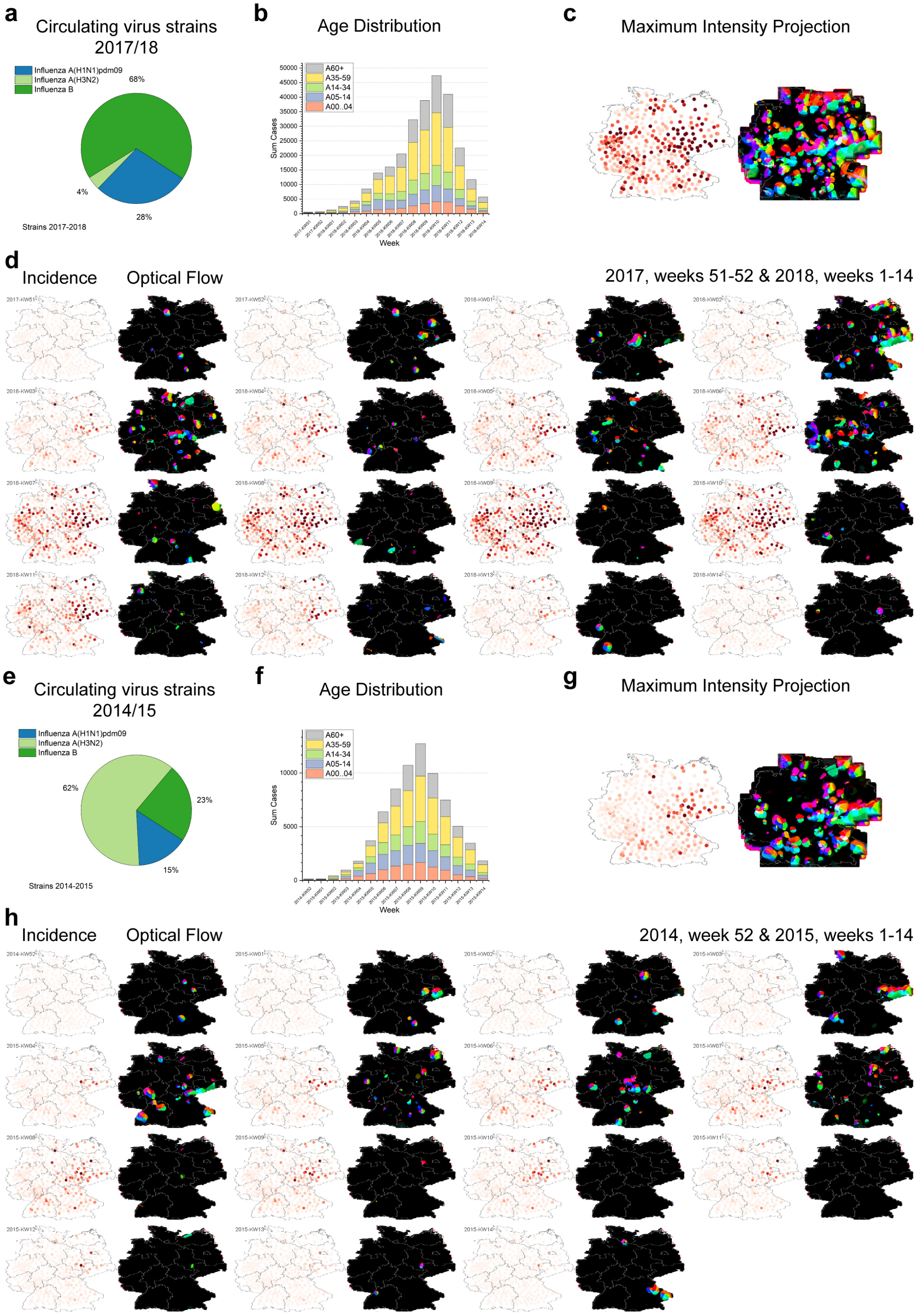

Figure S2: Analysis of the influenza outbreaks 2017/18 and 2014/15 in Germany. **(a, e)** Distribution of influenza virus strains in Germany as reported by the national sentinel system. **(b, f)** Age distribution of influenza cases. Of note, the number of infected people within the age group 35–59 years in 2017/18 was higher than during other influenza seasons in Germany. **(c, g)** Maximum intensity projection of influenza incidence and optical flow plots of selected weeks. **(d, h)** Tempo-spatial distribution of influenza incidence and optical flow signals. The pie and barcharts were created in Origin2019b[8].The federal state border overlay was created in SAGA Gis 7.6.1 [6] with publicly available data from the BKG Germany [5].Data source: [4], [3].

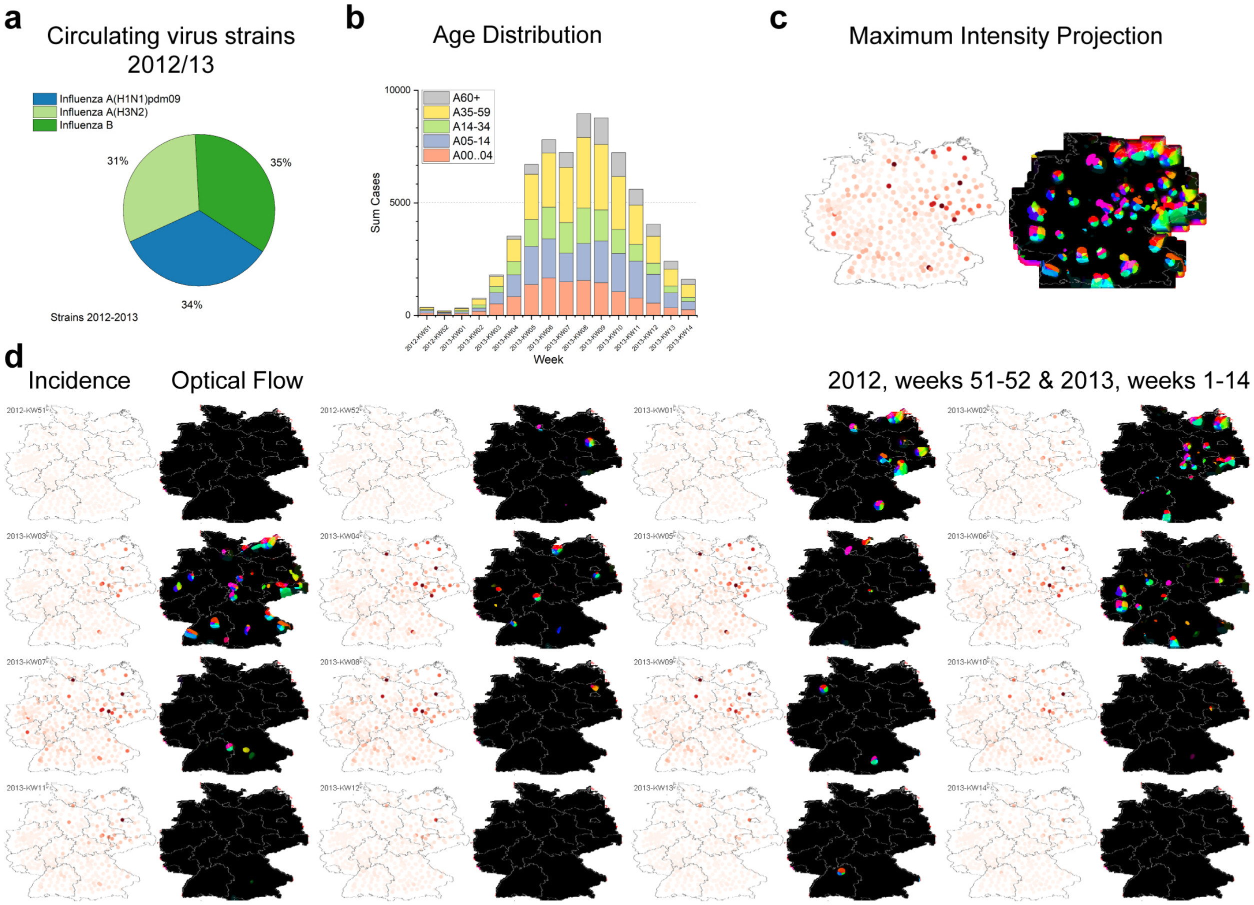

Figure S3: Analysis of the influenza outbreak 2012/13 in Germany. **(a)** Distribution of influenza virus strains in Germany as reported by the national sentinel system. **(b)** Age distribution of confirmed human influenza cases. **(c)** Maximum intensity projection of influenza incidence and optical flow plots of selected weeks. **(d)** Tempo-spatial distribution of influenza incidence and optical flow signals. The pie and barcharts were created in Origin2019b[8]. The federal state border overlay was created in SAGA Gis 7.6.1 [6] with publicly available data from the BKG Germany [5]. Data source: [2].

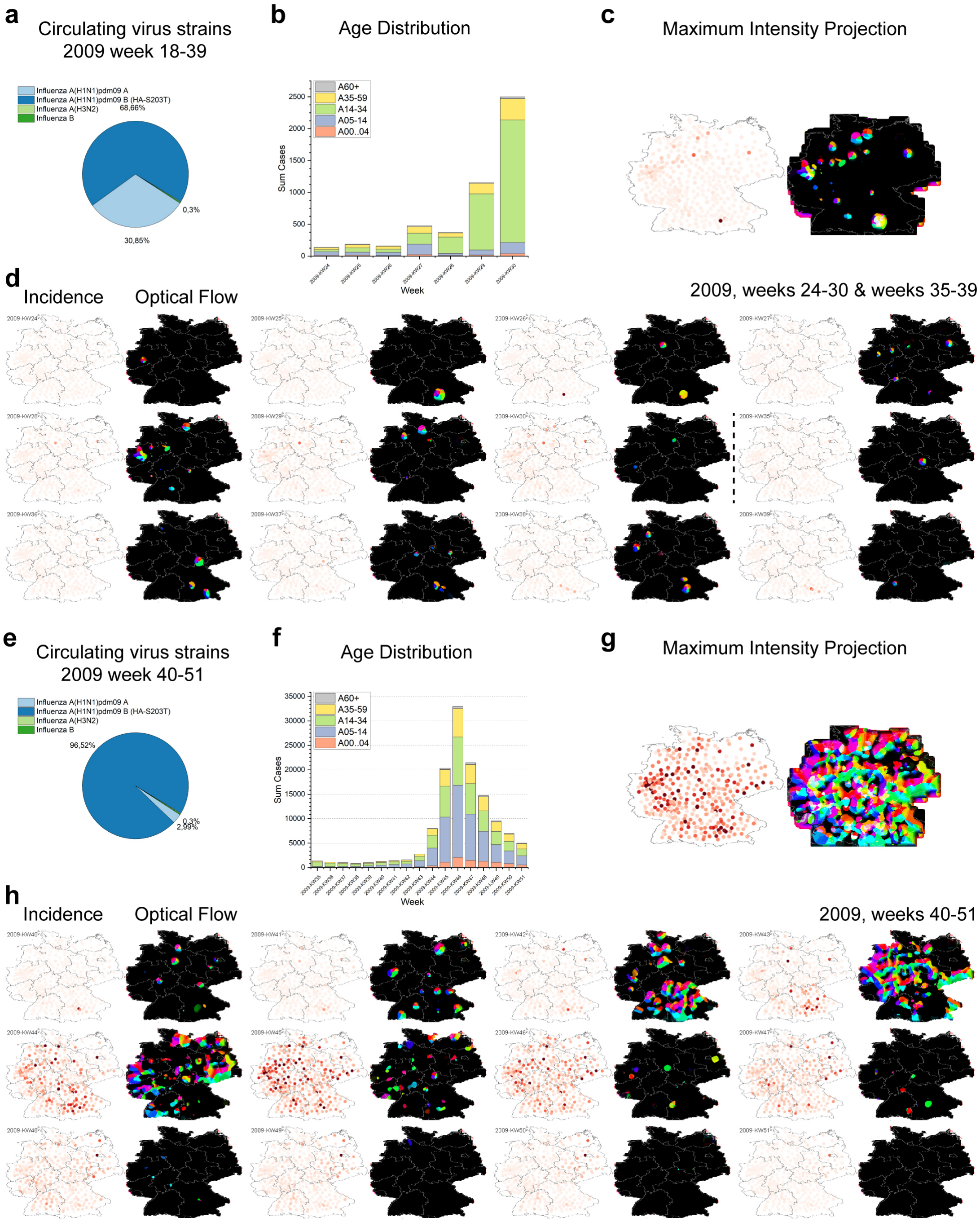

Figure S4: Analysis of the influenza outbreak 2009/10 in Germany. **(a)** Distribution of influenza virus strains in Germany in 2009 during calendar weeks 18–39 as reported by the national sentinel system. The pandemic strain influenza A(H1N1)pdm09 dominated (99.5%; split into group A (31%) and group B (69%)) over influenza A(H3N2 (0.2%) and influenza B (0.3%)). **(b)** Age distribution of confirmed human influenza cases. **(c)** Maximum intensity projection of influenza incidence and optical flow plots of selected weeks. **(d)** Tempo-spatial distribution of influenza incidence and optical flow signals. **(e)** Distribution of influenza virus strains in Germany in 2009 during calendar weeks 40–51. Group B of the pandemic strain influenza A(H1N1)pdm09 dominated over group A (97% vs. 3%). **(f)** Age distribution of confirmed human influenza cases. **(g)** Maximum intensity projection of influenza incidence and optical flow plots of selected weeks. In comparison to other influenza seasons the optical flow pattern during the second wave of the influenza A(H1N1) pandemic is covers much more parts of Germany. **(h)** Tempo-spatial distribution of influenza incidence and optical flow signals. The pie and barcharts were created in Origin2019b[8]. The federal state border overlay was created in SAGA Gis 7.6.1 [6] with publicly available data from the BKG Germany [5]. Data source: [1].

2 Supplementary Videos

Supplementary Video S1 The supplementary video S1 shows the tempo-spatial patterns of Noro and Influenza reported cases. Incidence intensity and location plots are shown on the left. Optical flow direction and intensity of the registered cases is shown on the right. For the decade of 2008-2018. Epidemical Data provided by Robert-Koch Institute Survstat 2019 [7]. Coordinate data was downloaded from BKG.de 2019[5].

Source: S1IncidenceOpticalFlowNoroInfluenca20082018.avi

Supplementary Video S2 The supplementary video S2 shows the tempo-spatial patterns of Noro and Influenza reported cases. Incidence intensity and location plots are shown on the left. Optical flow direction and intensity of the registered cases is shown on the right. For the decade of 2009-2010. Epidemical Data provided by Robert-Koch Institut Survstat 2019 [7]. Coordinate data was downloaded from BKG.De 2019[5].

Source: S2IncidenceOpticalFlowNoroInfluenca20092010.avi

References

[1] Robert-Koch-Institute (Germany). Bericht zur Epidemiologie der Influenza in Deutschland, Saison 2009/10. https://influenza.rki.de/Saisonberichte/2009.pdf, 2010.

[2] Robert-Koch-Institute (Germany). Bericht zur Epidemiologie der Influenza in Deutschland, Saison 2012/13. https://influenza.rki.de/Saisonberichte/2012.pdf, 2013.

[3] Robert Koch-Institute (Germany). Bericht zur Epidemiologie der Influenza in Deutschland, Saison 2014/15. https://influenza.rki.de/Saisonberichte/2014.pdf, 2015.

[4] Robert-Koch-Institute (Germany). Bericht zur Epidemiologie der Influenza in Deutschland, Saison 2017/18. https://influenza.rki.de/Saisonberichte/2017.pdf, 2018.

[5] BKG. Geobasis-de / bkg. *database* http://www.bkg.bund.de, 2019.

[6] O. Conrad, B. Bechtel, M. Bock, H. Dietrich, E. Fischer, L. Gerlitz, J. Wehberg, V. Wichmann, and J. Böhner. System for automated geoscientific analyses (SAGA) v. 2.1.4. *Geoscientific Model Development*, 8(7):1991–2007, July 2015.

[7] Robert-Koch-Institute (Germany). Survstat@rki 2.0. *database* https://www.rki.de/EN, 2019.

[8] OriginLab Corporation, Northampton, MA, USA. Origin(Pro) 2019b. *Website* https://www.originlab.com/, 2019.
